# Experience of Laparoscopic Cholecystectomy Under Spinal Anesthesia with Low-pressure Pneumoperitoneum - Prospective Study of 300 Cases

**DOI:** 10.4103/1319-3767.80385

**Published:** 2011

**Authors:** Manoranjan Kar, Jugal K. Kar, Bibhas Debnath

**Affiliations:** Department of Surgery, Medical College, Kolkata, India; 1 Department of Medicine, Midnapur Medical College, West Bengal, India; 2 Department of Surgery, Sreedurga Nurshing Home, Paschim Medinipur, India

**Keywords:** Cholecystectomy, gallstone disease, laparoscopic cholecystectomy, laparoscopy, low-pressure pneumoperitoneum, regional anesthesia, spinal anesthesia

## Abstract

**Introduction:**

Having long experience of open upper abdominal surgery under spinal anesthesia and laparoscopic cholecystectomy under general anesthesia, we performed this study of laparoscopic cholecystectomy with low-pressure pneumoperitoneum under spinal anesthesia to assess its safety and feasibility.

**Materials and Methods:**

In a private rural health set-up, 300 patients were selected prospectively for laparoscopic cholecystectomy under low-pressure (8 mm) pneumoperitoneum under spinal anesthesia in a span of three years. Only 3.5 ml of 0.5% bupivacaine was used for spinal anesthesia. Fourth port positioned at lower than usual at the level of umbilicus, change of position of the table with different stages of operation, massaging of right shoulder in cases of shoulder pain, removal of smoke if formed during dissection to diminish shoulder pain and holding the body of the gallbladder by the fourth port grasper at the level of lower margin of the liver in cases of long gallbladder were some modifications of standard laparoscopic cholecystectomy made in this study.

**Results:**

We successfully performed the operations in 291 patients without major complications. Four patients denied operation under spinal anesthesia. Spinal anesthesia was converted to general anesthesia in two patients due to severe shoulder pain. The operation was converted to open cholecystectomy in three patients. Mean age was 34.6 years (range 21-82 years). Mean BMI was –23.1 (range 20.8-28.3). Mean duration of operation was 39.6 min (range 18-78 min). Mean O_2_ saturation was 97.6%. Mean peak respiratory rate was 23.4 (range 16-38). 90.08% patients complained of right shoulder pain – most of them managed by shoulder massage alone. All patients were satisfied on follow up.

**Conclusion:**

Laparoscopic cholecystectomy under spinal anesthesia with low-pressure pneumoperitoneum can be performed safely and satisfactorily without major complications by experienced surgeons.

Having experience of more than five years of upper abdominal surgery under spinal anesthesia and experience of laparoscopic cholecystectomy under general anesthesia in rural health set-up, we started performing laparoscopic cholecystectomy under spinal anesthesia. The cost of upper abdominal surgery under spinal anesthesia is less than that under general anesthesia. We performed the operations using low-pressure pneumoperitoneum to avoid excess stretching of the diaphragm in conscious patients and to lower the complications rate of hypercarbia.

Certain technical points were modified to avoid complication.

This prospective study has been conducted to prove effectiveness and feasibility of spinal anesthesia for laparoscopic cholecystectomy in rural health set-up.

## MATERIALS AND METHODS

The study was conducted in a private peripheral rural surgery set-up from October 2006 to December 2009. First 300 patients were selected prospectively from the patients with gallstone diseases who opted for laparoscopic cholecystectomy.

Exclusion criteria of the patients were history of jaundice, previous history suggestive of cholangitis, acute cholecystitis, history of previous upper abdominal operations, raised level of serum alkaline phosphatase, Ultrasonographic features of edematous gallbladder wall, age less than 20 years, and suspicion of gallbladder malignancy.

Patients presenting with acute cholecystitis were managed conservatively and included for operation after three weeks. Four patients refused to accept spinal anesthesia for fear of low back pain. Ultimately 296 patients were operated under spinal anesthesia. Spinal anesthesia was converted to general anesthesia in two patients due to severe right shoulder pain and non-cooperation. All patients and patients’ relatives were informed in detail in the patient’s own language about the disease, types of operation possible, types of anesthesia available, cost of different types of operations and anesthesia, patients’ consciousness during operation, advantages and disadvantages of the operations, risk of conversion of anesthesia and operation and provision of live telecast of monitor’s view of the operation to patient’s relatives.

Initially, patients were admitted on the day before the operation and two tablets each of bisacodyl and festal were given on the night before the operation to lessen colonic distension. Per rectal enema was given in the morning hours on the day of operation. But later on, patients were admitted on the day of operation and operated in the evening.

Preoperatively about 500 ml of Ringer’s lactate solution was infused in all patients except patients with hypertension. Intravenous antibiotic (ceftriaxone) was given. Standard spinal puncture with 25G spinal needle was done between L1 and L2 spaces at right lateral position. Only 3.5 ml of 0.5% bupivacaine heavy was given. Patient was kept in right lateral position for about 30 sec and then turned to supine with head end of the table tilted down 10-15° with a pillow under the head.

Anesthesia level was checked with pinprick sensation. Patient was kept in this head down position for 6-8 min. Standard pulse-oxymeter was used for monitoring pulse and oxygen saturation. Blood pressure was monitored manually at 5 min interval. Any hypotension was managed with extra intravenous fluid infusion. Injection mephentermine (15 mg), single dose, per-operatively, was given intravenously in patients with systolic blood pressure below 80 mm of Hg even after adequate intravenous fluid infusion. Most of the patients were catheterized. Catheter was removed 12 h post-operatively. Specific proforma was used to note patient-related data, per operative findings and postoperative follow up. During operation, pulse rate, blood pressure, respiratory rate, oxygen saturation, shoulder pain, bleeding and any other difficulty was noted in a table format at 5 min interval.

After spinal puncture, when the patient was in head down position, antiseptic painting and draping, instrument set-up and standard umbilical ports were made. Open access umbilical ports and telescopic check of access before starting insufflations of CO_2_ pneumoperitoneum were done. Open umbilical ports were made in such a way that they fit 10 mm port without suturing. Intra-abdominal pressure was set at 8 mm of Hg. Pnemoperitoneum was started at the rate of 1 l/min. Now head end of the table was elevated and left tilt was made. Rate of insufflations was increased to 2 l/min. Then, the epigastric port was made and gallbladder pathology was checked. The third port (standard subcostal) at right mid-clavicular line was made. The fourth port was made at right mid-axillary line at the level of umbilicus.

We held the gallbladder to the body by the grasper through fourth port in cases where it was projecting beyond the liver margin significantly. Tense gallbladders were aspirated. Standard Callots’ triangle dissections were made. Cystic duct and artery were clipped proximally doubly. Distally cystic arteries were not clipped. Duct and artery were sectioned. Types of back bleeding from cystic artery stump were noted. If spurting type of back bleeding was noted, then distal cystic artery stump was held with Maryland dissector and coagulated. Further cautious dissection to search for another arterial supply of gallbladder was done and clipped if any. Now, after Callots’ dissection, elevation of head end of the table was lowered. Further dissections were made by electrocautery using hooks. Any irrigation and suction was made with head end of the table in flat position or down position with right tilt. Gallbladder was removed in the above noted table position through umbilical port. Enlargement of umbilical port was made by stretching with a large hemostat, or by cutting for removal of large size gallbladder. Initially, 50 cases were completed with placement of drain in the hepato-renal pouch in all the cases. Later on, we used drain selectively. The umbilical port’s sheath was repaired with 1-0 vicryl with taper-cut needle.

Monitor’s view of the operation was transmitted to a TV at the visitor’s rest room. Relatives of the patient also got the opportunity to see steps of operation. Patients also saw part of their operation in the monitor by rotating their head towards the monitor.

An OT sister always stood on the right side of the head end of the table, to assure the female patients and to massage the patients’ right shoulder if they complained of right shoulder pain. Some patients required intravenous injection of tramadol hydrochloride (100 mg) for unbearable shoulder pain even after massage. Excessive smoke formation during electrocautery aggravates right shoulder pain. So when there was smoke formation, we opened one port for exit of smoke and cleaned the electrocautery hook.

Operation time, post-operative pain, nausea, vomiting, headache, any neurodeficit, wound-related complications, ambulation time and patient satisfaction were noted.

Eight patients underwent laparoscopic appendicectomy and fourteen patients underwent laparoscopic tubectomy in addition to laparoscopic cholecystectomy after change of instrument- trolley position and making an extra port.

Patients were discharged after 72 h of operation, with removal of skin stitches. They were asked to come for first follow-up 15 days post-operatively and also advised to keep in touch over phone or through messenger.

## RESULTS

A total of 300 patients were included in our study. Two hundred and ninety-four patients underwent laparoscopic cholecystectomy under spinal anesthesia. Four patients did not give their consent for the spinal anesthesia. Among them three female patients having low back pain, thought of previous spinal anesthesia for LUCS was the causative factor for their low back pain. Another male patient refused fearing lower back pain in future. Seventy-nine patients were males; two hundred and twenty- one patients were females. Mean age was 34.6 years, age range from 21 years to 82 years. Body mass index was mean 23.1 (range 20.8-28.3). No morbid obese patient was present in the study [Table 1].

**Table 1:** Patients’ details

Total no of patients	300
No of patients rejected spinal anesthesia	04 (1.33)
Conversion of spinal to general anesthesia	02 (0.67)
Conversion of laparoscopy to open operation	03 (1.0)
Age – range 21years to 82 years	Mean age – 34.6 years
Sex – more female patients	M: F ratio – 1:2.83 (79:221)
BMI	Mean – 23.1, (range 20.8 to 28.3)
Concomitant operations following laparoscopic cholecystectomy	
Laparoscopic appendicectomy operations	08 (2.66)
Laparoscopic tubectomy operations	14 (4.66)
Mortality	Nil
Associated medical conditions	
COPD	Seven patients (2.34)
Hypertension	19 patients (6.34)
Diabetes mellitus	12 patients (4.0)
Heart block (RBBB)	Four patients (1.34)
Hypothyroidism on eltroxin	Six patients (2.0)
Obesity	Four patients (1.34)
Associated surgical conditions	
Upper abdominal scar	Two patients (0.67)
Inguinal hernia	One female patient (0.34)
Recurrent appendicitis	Eight patients (2.67)

Figures in parentheses are in percentage

Two patients were converted to general anesthesia due to severe right shoulder pain not responding to shoulder massage and injection tramadol hydrochloride and also non-cooperation during operations. Three patients needed conversion to open surgery. Among them, two patients were converted because of unclear anatomy. Third patient had mucocele of the gallbladder with fundus on left side of the falciform ligament and that was adherent with left lobe of liver. So ultimately, we completed the laparoscopic cholecystectomy operations in two hundred and ninety-one patients under spinal anesthesia.

The physician of our team controlled medical conditions and he assisted anesthetist during operations for management of any medical problem. Medical conditions associated with the patients were chronic obstructive pulmonary disease, hypertension, diabetes mellitus, incomplete heart block, hypothyroidism, and obesity. Two patients had upper abdominal scar, one due to previous truncal vagotomy and gastro-jejunostomy and the other due to laparotomy. One aged female patient had cholecystitis with large inguinal hernia, we performed laparoscopic cholecystectomy and open inguinal herniorrhaphy on her.

Eight patients underwent laparoscopic appendicectomy and fourteen patients underwent laparoscopic tubectomy in addition to laparoscopic cholecystectomy after change of instruments trolley position and making an extra port.

Two hundred and ninety one patients underwent laparoscopic cholecystectomy successfully under spinal anesthesia. No patient suffered total spinal anesthesia, respiratory arrest, and cardiac arrest during operations.

Most of the patients complained of pain at right shoulder tip. But they were managed by continuous finger massaging by an OT sister over the right shoulder area of the patients. Only twenty-six patients required injection tramadol hydrochloride plus massaging.

Oxygen saturation was maintained around 98% without oxygen supplementation. Respiratory rates were increased after peumoperitoneum that helped carbon dioxide washout through expirations. Maximum respiratory rate during pneumoperitoneum was 16 to 38 per min (mean 23.4 per min). During Callot’s dissection, patients were counseled to take quiet breaths. Most of the patients visualized their own operation in the monitor [Table 2].

**Table 2:** Anesthesia details

Spinal anesthesia	296 patients
Anesthesia: sensory loss (upper level)	T4 / T5 dermatome
Total spinal anesthesia	Nil
Respiratory arrest	Nil
Cardiac arrest	Nil
Intra-abdominal pressure	8 mm of Hg
Shoulder pain	262 patients.
Managed by right shoulder massage alone	236 patients (90.08)
Managed by massage plus inj. tramadol (100 mg)	26 patients (9.93)
Oxygen saturation	96-99% (mean.97.6)
Respiratory rate	
Basic (before pneumoperitoneum)	13-16 (mean. 14.3)
Maximum (with pneumoperitoneum)	16-38 (mean.23.4)
Hypotension (systolic below 100 mm of Hg)	36 patients (12.00)
Managed by IV fluid only	29 patients
Managed by IV fluid plus inj mephentermine	7 patients
Hypertensive episodes	Nil
Post-spinal headache	Five patients (1.66)
Nausea/ vomiting	22 patients (7.34)
Low back pain (temporary)	Five patients
Neurological deficit	Nil

Figures in parentheses are in percentage

Blood pressure was maintained at normal range but thirty-six patients developed hypotensive episodes. Twenty-nine patients were managed with extra intravenous fluid alone. Seven patients needed injection mephentermine 15 mg, intravenously, single dose per-operatively along with I V fluid. In the post-operative period, foot-end of the patient’s bed was kept five inches elevated for 4 h in every patient. No patient developed hypertensive episodes because of spinal anesthesia and position of the patients during operations.

Five patients complained of post-spinal headache, especially where first spinal puncture was unsuccessful. Nausea and vomiting occurred in post-operative period in those patients who required longer operation time. Sublingual ondansetron tablets were used to manage nausea.

Some complained of low back pain temporarily, but that disappeared within two weeks. No neurodeficit was noted in any patients

We completed laparoscopic cholecystectomy under spinal anesthesia in 291 patients. The duration of operations (skin incision to skin closure) was 18 min to 78 min (mean 39.6 min). The elongated gallbladders were managed by our own technique by holdings it with second grasper (through forth port) at lower margin of the liver during dissection. In thirteen patients, wall of gallbladder was perforated during dissections by diathermy hook. We irrigated the sub-hepatic and sub-diaphragmatic area with normal saline in cases of bile spillage after gallbladder perforation and also in cases of hemorrhage [Table 3].

**Table 3:** Operations details

Laparoscopic cholecystectomy under spinal anesthesia	291 patients
Duration	18 to 78 min (mean 39.6 min)
Gut injuries	Nil
Adhesions (gallbladder)	84 patients
Large Hartman’s pouch	38 patients
Gallbladder projecting beyond liver margins in excess of 5cm	17patients
Gallbladder perforations (during dissection)	13 patients
Gallstone spillage	Six patients
Bile duct injuries	Nil
Post-op bile leakage	Nil
Peritonitis	Nil
Jaundice	Nil

Twelve patients complained of mild umbilical wound discharge on first follow-up. The wound healed up spontaneously on dressing.

All the patients were satisfied with results of operations on follow up. No patient complained against any step of anesthesia or the operation during follow up.

## DISCUSSION

Laparoscopic cholecystectomy is the gold standard for treatment of uncomplicated symptomatic cholelihiasis. General anesthesia is regarded as safe anesthesia for laparoscopic surgery in most of the cases till now. We have long-term experience of performing upper abdominal surgery under spinal anesthesia in this rural setup and experience of performing laparoscopic cholecystectomy under general anesthesia.

Single puncture spinal anesthesia can be an easier technique than general anesthesia.[1] Monitoring of patients under spinal anesthesia is easier than general anesthesia. Complication of endotracheal intubations like damage to oral cavity, teeth, sore throat, and aspirations, failure of intubations are absent in spinal anesthesia. Cost of spinal anesthesia is far less than general anesthesia. Nausea and vomiting are less with spinal anesthesia.[1–13] Laparoscopic cholecystectomy with low-pressure pneumo-peritoneum under spinal anesthesia is effective in patients with COPD, who are unfit for general anesthesia.[5,6]

The patients with acute cholecystitis visited our clinic four to five days after the onset of pain, and had got empirical treatment from the local doctor during this time. Most of them suffered from recurrent attack of acute cholecystitis before attending the clinic. We expected fibrosed and edematous Callot’s triangle in those patients. For this reason, we excluded the patients during acute cholecystitis from this study.

Initially, we catheterized all patients to avoid urinary retention in the postoperative period due to extra IV fluid infusion during spinal anesthesia. But after operation of about 50 patients, we infused optimum amount of intravenous fluid. Later on, we catheterized the patients selectively where amount of infusion of fluid was more than 1500 ml per-operatively or duration of operation was more than 50 min.

Hypotension is a problem of spinal anesthesia, which can be overcome by preloading with fluids.[7,13] Laparoscopic surgery under general anesthesia is associated with hypertensive episodes; but under spinal anesthesia, there were no such episodes of hypertension in any patients.

Injection mephentermine was used in single dose of 15 mg, intravenously. Usually within 5-10 min of elevation of the head end of the table during the operation, a single dose was used only in those patients where systolic blood pressure fell below 80 mm of Hg despite adequate fluid infusion.

Lowering of head end of table after Callots’ triangle dissection, elevation of foot end of the table during repair of the ports and during postoperative period, also carbon-di-oxide pneumoperitoneum prevent fall of blood pressure.

Patients were fully conscious during operations under spinal anesthesia. Respiration rate was increased to washout carbon-di-oxide.[1,2] Here, as we operated under low-pressure (8 mm) pneumo-peritoneum, incidences of hypercarbia producing hypertensive episodes were negligible. Vasodilatations under spinal anesthesia and elevated head end of the patient were also preventive against hypertensive episodes during operation.[1,2]

Per-op shoulder pain can be managed by finger massaging over right shoulder, diminution of elevation of head end of the table after Callot’s dissection, using low-pressure pneumoperitoneum and clearing-out of smoke due to diathermy at the earliest. Lower rate of carbon-di-oxide inflow (1-2 L) during initial phase of inflation can avoid shoulder pain. Post-op shoulder pain can be avoided by near complete evacuation of pneumoperitoneum and by elevation of foot end of the table.

Standard dissection of cholecystectomy using four ports under low-pressure pneumoperitoneum is not difficult as compared to laparoscopic cholecystectomy under general anesthesia with high-pressure pneumoperitoneum. In cases of long size gallbladder which are projecting beyond liver margin by more than 5 cm, laparoscopic cholecystectomy was performed by holding body of the gallbladder at the lower margin of liver.[12] Low-pressure pneumoperitoneum and spinal anesthesia cause no problem for space during operation.

Complications of cholecystectomy are similar to standard laparoscopic cholecystectomy under general anesthesia.

## CONCLUSION

Laparoscopic cholecystectomy under spinal anesthesia with low-pressure pneumoperitoneum by experienced surgeons is safe and cost-effective, associated with minimal postoperative pain and smooth recovery.
